# The Use of Immersive Virtual Reality (VR) to Predict the Occurrence 6 Months Later of Paranoid Thinking and Posttraumatic Stress Symptoms Assessed by Self-Report and Interviewer Methods: A Study of Individuals Who Have Been Physically Assaulted

**DOI:** 10.1037/a0036240

**Published:** 2014-04-07

**Authors:** Daniel Freeman, Angus Antley, Anke Ehlers, Graham Dunn, Claire Thompson, Natasha Vorontsova, Philippa Garety, Elizabeth Kuipers, Edward Glucksman, Mel Slater

**Affiliations:** 1 Department of Psychiatry, University of Oxford; 2 Department of Experimental Psychology, University of Oxford; 3 Centre for Biostatistics, Institute of Population Health, Manchester University; 4 Department of Psychology, Institute of Psychiatry, King’s College London; 5 Emergency Department, King’s College Hospital, London, United Kingdom; 6 Department of Computer Science, University College London, and Institució Catalana de Recerca i Estudis Avançats (ICREA), University of Barcelona

**Keywords:** virtual reality (VR), paranoia, posttraumatic stress disorder (PTSD)

## Abstract

Presentation of social situations via immersive virtual reality (VR) has the potential to be an ecologically valid way of assessing psychiatric symptoms. In this study we assess the occurrence of paranoid thinking and of symptoms of posttraumatic stress disorder (PTSD) in response to a single neutral VR social environment as predictors of later psychiatric symptoms assessed by standard methods. One hundred six people entered an immersive VR social environment (a train ride), presented via a head-mounted display, 4 weeks after having attended hospital because of a physical assault. Paranoid thinking about the neutral computer-generated characters and the occurrence of PTSD symptoms in VR were assessed. Reactions in VR were then used to predict the occurrence 6 months later of symptoms of paranoia and PTSD, as assessed by standard interviewer and self-report methods. Responses to VR predicted the severity of paranoia and PTSD symptoms as assessed by standard measures 6 months later. The VR assessments also added predictive value to the baseline interviewer methods, especially for paranoia. Brief exposure to environments presented via virtual reality provides a symptom assessment with predictive ability over many months. VR assessment may be of particular benefit for difficult to assess problems, such as paranoia, that have no gold standard assessment method. In the future, VR environments may be used in the clinic to complement standard self-report and clinical interview methods.

An immersive virtual reality (VR) system creates a surrounding three-dimensional, computer-generated world in which a person can physically move and interact with objects and virtual people. The laboratory room becomes replaced—typically via a tracked headset worn by the person—by an alternate digitally created and computer-generated world. The movements of participants are tracked in real time, so the images are visually updated as a function of head gaze position and orientation. The interest derives from the fact that VR scenes elicit responses in individuals similar to those that would occur in the real situation (Sanchez-Vives & Slater, 2005). For example, virtual reality heights are as effective as exposure to actual heights in the treatment of acrophobia (e.g., Emmelkamp et al., 2002). VR has also been successfully used in the treatment of posttraumatic stress disorder (PTSD) arising from, for example, combat (e.g., Rothbaum, Hodges, Ready, Graap, & Alarcon, 2001) or a road traffic accident (e.g., Beck, Palyo, Winer, Schwagler, & Ang, 2007). Indeed, virtual reality exposure therapy has been shown to have large effect sizes in the treatment of anxiety disorders (see the meta-analysis by Powers & Emmelkamp, 2008). In addition, a key advantage of the technology is that it has the potential to provide standardized, ecologically valid tests of symptom occurrence in the here and now. The use of VR may assist clinicians’ judgments. In the future, clinical assessment may no longer rely solely on retrospective patient past recall or interviewer questioning but may include observation of actual reactions in VR environments.

Our particular interest has been to use VR to study paranoia, which comprises a spectrum of severity of ideas of reference and persecution. Persecutory ideation, the severer form of paranoia, is defined as unfounded or excessive thoughts that others deliberately intend harm to the person (Freeman & Garety, 2000). The difficulty with studying paranoia, particularly in its milder forms in the general population, is determining that the thoughts about hostility are unfounded. This has been labeled the “paranoia problem” (Freeman, 2008a). When asking about paranoid thoughts, a clinical interviewer must judge whether the thoughts at the time were excessive or unfounded; this can be difficult, because the interviewer was not in the situation in which the suspicious thoughts occurred. The person may actually have been, for example, the victim of hostile words or a threatening stare, or he or she may have been intentionally pushed aside. In essence, environments can be hostile and people are persecuted. Furthermore, the interviewer judges these events from the person’s retrospective recall of past weeks. Therefore it is highly likely that inaccuracy in the assessment of paranoia is introduced both by the inclusion of thoughts that are realistic and by the limitations of past recall. We have therefore developed VR as a means of assessing the occurrence of unfounded paranoid thoughts without relying on retrospective recall. By presenting VR social situations that are neutral, one can assess whether an individual is misinterpreting the computer characters (avatars) as being hostile. Moreover, the person cannot act in a way that will elicit hostile reactions from the avatars. It is therefore an ideal way to assess paranoia. We have used VR to study the spectrum of paranoia severity, including in individuals with nonclinical paranoia (Freeman et al., 2003, 2005, 2008; Freeman, Evans, et al., 2013), those at high risk of psychosis (Valmaggia et al., 2007), and patients with schizophrenia (Freeman, Pugh, Vorontsova, Antley, & Slater, 2010). There is no gold standard assessment of paranoia because of the difficulties of determining the accuracy of the thoughts; VR has the potential to become an important part of the setting of such a standard.

Individuals who report having paranoid thoughts about the avatars also report such fears in everyday life (e.g., Freeman et al., 2008), providing evidence of convergent validity for the VR assessment scores. Freeman et al. (2010) found that patients with persecutory delusions have greater levels of paranoia in VR scenarios than individuals with high nonclinical paranoia, who in turn have higher levels of paranoia in VR than individuals with low levels of paranoia. This finding demonstrated criterion validity for test scores. There is evidence of test–retest reliability: Repeating the VR scene on the same day produces similar test scores (Freeman et al., 2007). Moreover, a theoretical model of persecutory delusions has proved successful in predicting the occurrence of paranoia in the VR environment (e.g., Freeman et al., 2005). However it is unknown whether having paranoid thoughts in VR is predictive of having paranoid thoughts in the future (predictive validity). The previous studies have shown the potential of VR for use in the clinic for the assessment of current paranoia, but further validity would be added if VR were also a predictor of the persistence of such a thinking style over time.

We have recently reported on a cohort in which the detection of true paranoid thinking is particularly difficult: individuals in the general population who have been recently physically assaulted (Freeman, Thompson, et al., 2013). Rates of paranoia are substantially raised in people with trauma and PTSD (e.g., Campbell & Morrison, 2007; Gracie et al., 2007); for example, in a national epidemiological sample, people with a probable diagnosis of PTSD had over 25 times the odds of also reporting fears of plots against them (Freeman et al., 2011). In the cohort study, virtual reality was included in the initial assessment battery that was administered 4 weeks after the assault, in order to validate the baseline scores on the study’s main interviewer and self-report paranoia assessments (Freeman, Thompson, et al., 2013). We wanted to see whether fears would generalize to the computer avatars (i.e., whether the fears had become demonstrably excessive). It was found that those people who had paranoid thoughts in VR were reporting greater levels of paranoia in everyday life. The participants were then tested again on the standard paranoia measures 6 months later. In this paper we report for the first time on the ability of the responses in VR to predict the occurrence of paranoia as assessed by standard self-report and assessor-rated measures at the 6-month follow-up. It was predicted that paranoia in the neutral VR social environment would be associated with standard symptom scores at follow-up. Associations were expected to be modest, because each assessment method has its own strengths and weaknesses in assessing paranoia. VR has ecological validity, but in the current study only one social environment was presented to the participants; self-report and interviewer methods can assess multiple situations, but these methods rely on past recall and can capture instances of true hostility.

The opportunity was also taken to assess the occurrence of PTSD symptoms in VR as predictors of later posttraumatic symptoms. Although VR has been a method of treatment delivery for PTSD, it has not been used as an assessment tool for this condition. PTSD assessments do not typically have the same difficulty of determining the evidential basis of the symptoms, but responses to VR situations may still add useful information to self-reports and clinical interviews. For example, such responses may be helpful when it is unclear whether the individual’s concerns about risk are exaggerated or when symptoms may be underreported because the individual does not link them to the trauma (e.g., emotional and physiological responses to trauma reminders may be missed if the patient does not recognize the triggers). In the first such test, we predicted that PTSD symptoms elicited in VR at 1 month after an assault would be associated with PTSD assessment scores obtained 6 months later.

## Method

### Participants

Over the course of a year, 106 individuals were recruited to the study. The inclusion criteria were as follows: experienced a distressing assault within the previous month; attended the Accident and Emergency Department at King’s College Hospital, London, for related injuries; was age 18 to 65; and could attend a baseline assessment between 4 and 6 weeks after the assault. Most assaults happened far from home (*n* = 74), close to home (*n* = 26), or, more rarely, at home (*n* = 6). The main exclusion criteria were (a) the assault was part of ongoing abuse; the individual (b) had a history of diagnosed severe mental illness (schizophrenia or bipolar disorder); (c) had been diagnosed with alcohol or drug dependence; or (d) had insufficient command of English, so the assessments could not be completed. Ninety-four participants completed the follow-up assessment 6 months later. Further details of the recruitment process are provided in Freeman, Thompson, et al. (2013).

### Assessments

The socioeconomic classification of the participants was carried out with the occupationally based National Statistics Socio-economic Classification (ND-SEC) analytic classes (Office for National Statistics, 2005).

#### Paranoia

##### Positive and Negative Symptom Scale (PANSS; Kay, 1991)

In the current report the PANSS Suspiciousness item was only used as the interviewer-rated level of paranoia. This item is rated for the past week on a 1 (*absent*) to 7 (*extreme*) scale. Twelve tapes of assessments were re-rated, and there was high interrater reliability for the PANSS positive subscale score (intraclass correlation coefficient = .93).

##### Green et al. Paranoid Thoughts Scale (GPTS; Green et al., 2008)

The GPTS is a 32-item self-report scale assessing the occurrence of ideas of reference and persecution. Each item is rated on a 1–5 scale. The presence of persecutory ideation is assessed over the past month, and higher scores indicate greater levels of paranoia. The internal reliability of the scale items was very high (baseline Cronbach’s alpha = .98; 6-month follow-up Cronbach’s alpha = .98).

##### VAS Paranoia

A sum from four self-report visual analogue 0–100 scales (VAS) was also used to assess paranoia (“Since the assault, I feel suspicious of other people”; “Since the assault, I feel fearful of all males”; “Since the assault, I feel fearful of all females”; “Since the assault, I feel more fearful of other people than I should”). The internal reliability of the combined scale items was high (baseline Cronbach’s alpha = .83; 6-month follow-up Cronbach’s alpha = .90).

#### PTSD

##### Interviewer version of the PTSD Symptom Scale (PSSI; Foa, Riggs, Dancu, & Rothbaum, 1993)

The PSSI is a 17-item scale assessing symptoms over the past fortnight of reexperiencing, avoidance, and arousal. It is rated by the interviewer. Higher scores indicate higher levels of PTSD symptoms. The internal reliability of the scale was high (baseline Cronbach’s alpha = .91; 6-month follow-up Cronbach’s alpha = .92). Twelve tapes of assessments were re-rated, and there was very high interrater reliability for the PSSI total score (intraclass correlation coefficient = .99). The PSSI performs similarly to the Clinician-Administered PTSD Scale (CAPS; Foa & Tolin, 2000).

##### Posttraumatic Diagnostic Scale (PDS; Foa, Cashman, Jaycox, & Perry, 1997)

The PDS is a self-report scale comprising 17 items assessing over the past month symptoms of reexperiencing, avoidance, and arousal. Higher scores indicate higher levels of PTSD symptoms. The internal reliability of the scale items was very high (baseline Cronbach’s alpha = .91; 6-month follow-up Cronbach’s alpha = .95).

#### Virtual reality

The VR procedure was identical to that used by Freeman et al. (2008). The head-mounted display was a Virtual Research VR1280, which has a resolution of 1280 × 1024 in each eye, a 60° diagonal field of view, and a refresh rate of 60 Hz. The tracking system was the Intersense IS900. The tracker uses a hybrid of inertial and ultrasonic sensors to determine the orientation and position of the user during the simulation. The sensors were laid out in a ceiling constellation grid above the user, who could freely walk around. The virtual reality environment comprised a 4-minute journey between two stops on a London underground (“tube”) train, populated by avatars. The underground train system is a key well-used public transport system in London. The Distributed Immersive Virtual Environment (DIVE) software platform was used to create the overall scenario (Frécon, Smith, Steed, Senius, & Stahl, 2001). Both the train shell and the avatars were created with 3D Studio Max run on Windows. The avatar motions were made with an optical motion capture system. Each avatar had its own background motion that repeated throughout the scenario. Each avatar had one motion that approximated its breath and another motion that randomized the direction of its gaze. In addition, several of the avatars responded to participants’ gaze by looking in their direction (e.g., one avatar would occasionally smile at the user when looked at). Ratings indicate that most members of the general public view the avatars as neutral or friendly (Freeman et al., 2008). The audio for the scene, comprising background tube noise and low-level snippets of conversation, was rendered in stereo, without spatialization, using a Creative sound card. After completing the journey, participants completed the following assessments:

##### State Social Paranoia Scale (SSPS; Freeman et al., 2007)

The SSPS was specifically designed to assess paranoia in VR. It comprises 10 persecutory items (e.g., “Someone stared at me in order to upset me”; “Someone was trying to isolate me”; “Someone was trying to make me distressed”), each rated on a 5-point scale. Higher scores on the scale indicate greater levels of persecutory thinking. The internal reliability of the scale items in the current study was high (Cronbach’s alpha = .87).

##### VR PTSD

We assessed PTSD symptoms during the virtual reality train ride with a newly constructed 13-item self-report scale, adapted from items that could be applied to a state measure in existing PTSD measures such as the PDS, assessing re-experiencing (e.g., “Upsetting thoughts or images about the assault came into my head when I didn’t want them to”), avoidance (e.g., “I tried not to think about or have feelings about the assault”), and arousal (e.g., “I felt jumpy or easily startled (for example, by sudden noises)”). The internal reliability of the scale items in the current study was high (Cronbach’s alpha = .89).

##### Visual analogue rating scales

Participants also completed four VAS items, each rated on separate 10-cm lines. The first item was the degree to which the people on the train were hostile (from not hostile to extremely hostile); the second item was how paranoid they felt (from not paranoid to strongly paranoia); the third item was the degree to which the environment brought back memories, thoughts, or feelings about the assault (from did not remind to reminded very much); and the final item assessed the degree of presence in the scene (“Which was strongest on the whole, your sense of being in the real world of the laboratory or being on the virtual tube?”; rated from being in the laboratory to being in the virtual tube). Each of these items was used separately, with higher ratings indicating greater endorsement of the characteristic.

### Design

The study received approval from a National Health Service Research Ethics Committee. The initial assessment was completed 4 weeks after the assault. Participants completed a detailed battery, including the VR assessment. The 6-month follow-up was much briefer, comprising only the three paranoia and two PTSD interviewer and self-report assessments. Two postgraduate psychologists completed the ratings during the study (including the re-rating of tapes).

### Analysis

Analyses were carried out with SPSS Version 19.0. In the key test, univariate linear regression was used to determine to what extent the VR symptom scores predicted the 6-month scores obtained from the standard paranoia and PTSD assessments. For comparison, parallel univariate analyses were also used to test the prediction of 6-month scores from the corresponding baseline standard symptom measure (taken at 4 weeks after the assault). Finally, in a multivariate analysis predicting the 6-month standard symptom scores, the VR symptom score and the initial score for the respective symptom measure were simultaneously entered into linear regressions (using the Enter method). Significance test results for all the analyses are quoted as two-tailed probabilities.

## Results

### Demographics

The demographic details for the participant group are presented in Table 1. As would be expected for a physical assault group, there were a greater number of male participants than female, and the mean age was relatively young.[Table-anchor tbl1]

### Paranoia and PTSD at 4 Weeks After the Assault

The participants generally rated they felt more present in the virtual tube than the laboratory room (mean sense of presence score = 5.6, *SD* = 3.5). Paranoia in VR (SSPS) positively correlated with visual analogue scales for how hostile the participants thought the people on the train were (*r* = .52, *p* < .001) and how paranoid they felt on the train (*r* = .46, *p* < .001). Paranoia in VR correlated with the interviewer and self-report measures of paranoia taken at 4 weeks after the assault, PANSS Suspiciousness (*r* = .25. *p* = .010), GPTS total score (*r* = .34, *p* < .001), and paranoia VAS score (*r* = .29, *p* = .002). The interviewer assessment of paranoia (PANSS Suspiciousness) positively correlated with the GPTS (*r* = .66, *p* < .001) and paranoia VAS score (*r* = .54, *p* < .001).

PTSD symptoms in VR positively correlated with a visual analogue scale for how much the tube journey brought back memories, thoughts, or feelings about the assault (*r* = .59, *p* < .001). PTSD symptoms in VR correlated with interviewer-rated and self-reported PTSD symptoms at 4 weeks after the assault, PSSI score (*r* = .67, *p* < .001), and PDS score (*r* = .64, *p* < .001). Interviewer (PSSI) and self-report (PDS) PTSD assessments were highly correlated (*r* = .89, *p* < .001).

### The Prediction of Paranoia and PTSD 6 Months Later

The main tests, the associations of VR scores for paranoia and PTSD at 4 weeks with later interviewer and self-report assessments at 6 months, are shown in Tables 2 and 3. VR paranoia predicted subsequent paranoia scores (β between .37 and .43), and VR PTSD symptoms predicted subsequent PTSD scores (β between .49 and .58). Additionally, we tested whether VR responses predict over and above initial scores on the same outcome measures (see Tables 2 and 3). VR paranoia explained variance over and above interviewer and self-reported paranoia. When VR paranoia and PANSS Suspiciousness at 4 weeks were used to predict 6-month PANSS Suspiciousness, 42% of the variance was explained; when baseline PANSS Suspiciousness alone was used as the predictor, 36% of the variance was explained. Similarly, 9% additional variance was added to the prediction of the VAS Paranoia score at 6 months by including VR paranoia in addition to the initial VAS Paranoia score; however, only 1% variance was added by the inclusion of VR paranoia for the prediction of the GPTS score. For PTSD symptoms, VR PTSD predicted interviewer-rated PTSD symptoms at 6 months over and above interviewer-rated PTSD at 4 weeks but not for self-reported PTSD symptoms. VR PTSD and PSSI baseline score explained 49% of the variance in 6-month PSSI scores, and PSSI baseline score alone explained 46% of the variance.[Table-anchor tbl2][Table-anchor tbl3]

## Discussion

Seven applications of virtual social environments to schizophrenia have been set out (Freeman, 2008b): symptom assessment, identification of symptom markers, establishment of predictive factors, tests of putative causal factors, investigation of the differential prediction of symptoms, determination of toxic elements in the environment, and development of treatment. This study concerned symptom assessment. It is the first study to examine the ability of symptom occurrence in a situation presented briefly via immersive virtual reality to predict the later occurrence of psychiatric symptoms. Paranoid thoughts and PTSD symptoms were both assessed for a 4-minute VR train ride. A train ride is an appropriate scenario because a train is a commonly used public place where other people are present. The occurrence of these problems in VR predicted interviewer and self-report standard assessments both concurrently and 6 months later. This suggests that VR may be a useful tool in assessment, especially in cases where it is unclear whether the patient’s fears are unfounded.

Responses to a single, brief VR assessment correlated with concurrent self-reported and interviewer-rated paranoia and PTSD symptoms and also predicted self-report and interviewer-rated paranoia and PTSD symptoms 6 months later, suggesting that the different methods assess related phenomena. Moreover, responses to VR explained additional variance of symptoms at 6 months over and above the initial standard interviewer assessments, as well as for self-reported paranoia (but not to self-reported PTSD symptoms). It would of course be expected that a baseline symptom measure would be the strongest predictor of the same measure repeated at a later date, but it is noteworthy that the behavioral data provided by the VR method added to the accuracy of prediction.

The results have possible implications for the assessment of paranoia. A gold standard for the assessment of paranoia has not been established, given obvious problems of both interviewer and self-report methods. This is reflected in only moderate correlations between the self-report and interview-based paranoia measures in this study. VR methods, when suitably developed, have the potential for greater accuracy and objectivity than other approaches that rely mainly on self-report and clinical judgments, as they allow an unambiguous assessment of whether the individual’s negative thoughts about other people are unfounded. Our view is that VR will eventually form a key component of a rigorous assessment of paranoia and will complement self-report recall and clinical clarificatory cross-questioning. Associations between the VR and other paranoia measures were only modest, but this was expected because of the limitations in each of their accuracies. VR has the important advantage that it clearly assesses unfounded paranoid thoughts, but it relied in this instance on a single presentation of a scenario. Interviewer and self-report methods have the advantage that they cover many situations, but they have the difficulties of relying on past recall and assessing whether thoughts were unrealistic. Thus, VR responses to a range of relevant situations could ideally be used to increase the accuracy of paranoia assessments.

Determining the unfounded nature of thoughts is less central to the assessment of PTSD, which will explain why the VR assessment added less to the interviewer methods. However, it should be remembered that a VR environment tailored for the environment in which the person had been assaulted was not used, as this would have limited the accuracy of this PTSD assessment.

A key limitation of the study is that only one virtual environment was used. A train ride may not be a relevant elicitor of symptoms for some participants. Interviewer and self-report methods do not depend on fears occurring in only one circumstance. Greater accuracy would be obtained by presentation in VR of several common situations. This could include gradation of difficulty of each. For future research it is interesting to note that the equipment and programming that were used in the current study were from 2006. There have been significant improvements in the technology in the intervening period. For example, current head-mounted displays have much wider field of views, the wearing of an optical motion capture suit can be used to give participants a virtual body, and rendering of environments, especially people, is much more realistic (Freeman, Evans, et al., 2013). There is also the possibility for greater nuance in the presentation of avatars, in terms of facial expressions, eye contact, and reactions in response to participants. We would expect to see even greater accuracy in symptom assessment as immersion in an environment increases (Slater, 2009). Further, the cost of VR equipment is rapidly decreasing, enabling the possibility of widespread use in clinics. This will be contingent upon the availability of a greater choice of VR environments and tests of the predictive abilities of different degrees of immersion. Overall, the current study indicates that there is clear potential for VR to add to the accuracy of assessment of a number of mental health problems.

## Figures and Tables

**Table 1 tbl1:** Demographic Information

Variable	Participants (*N* = 106)
Mean age (*SD*)	34.4 (11.6)
Gender (*n*)	
Male	79
Female	27
Ethnicity (*n*)	
White	55
Black Caribbean	14
Black African	15
Black other	5
Other	17
Marital status (*n*)	
Single	81
Married	19
Divorced/separated	6
Education (*n*)	
None	11
GCSE	23
AS/A-level	9
Diploma/foundation degree	23
Degree	26
Postgraduate diploma	11
Doctorate	3
Socioeconomic status (*n*)	
Large employers and higher managerial	3
Higher professional	9
Lower managerial and professional	15
Intermediate	10
Small employers and own account	11
Lower supervisory and technical	6
Semi-routine	10
Routine	12
Long-term unemployed	15
Students	13
*Note*. Age is in years. GCSE = General Certificate of Secondary Education; AS/A-level = Advanced Subsidiary/Advanced level.	

**Table 2 tbl2:** The Prediction of Paranoia Scores at 6 Months From VR Response and Symptom Measures at 4 Weeks

Independent variable	PANSS Suspiciousness (interviewer)				GPTS Paranoia total (self-report)				VAS Paranoia total (self-report)			
	Unstandardized coefficient		Standardized coefficient	*p*	Unstandardized coefficient		Standardized coefficient	*p*	Unstandardized coefficient		Standardized coefficient	*p*
	*B*	Standard error			*B*	Standard error			*B*	Standard error		
1. Univariate predictors entered in regression												
VR Paranoia (alone)	0.08	0.02	.43	<.001	1.71	0.45	.37	<.001	6.93	1.52	.43	<.001
Initial score for outcome measure (alone)	0.61	0.08	.61	<.001	0.66	0.07	.71	<.001	0.52	0.09	.54	<.001
2. Predictors entered simultaneously												
VR Paranoia (allowing for initial outcome measure score)	0.05	0.02	.28	.001	0.62	0.07	.13	.094	5.00	1.38	.31	<.001
Initial score for outcome measure (allowing for VR Paranoia)	0.53	0.08	.53	<.001	0.62	0.07	.66	<.001	0.44	0.08	.46	<.001
*Note.* VR = virtual reality; PANSS = Positive and Negative Symptom Scale; GPTS = Green et al. Paranoid Thoughts Scale; VAS = visual analogue.												

**Table 3 tbl3:** The Prediction of PTSD Scores at 6 Months From VR Response and Symptom Measures at 4 Weeks

Independent variable	PSSI total (interviewer)				PDS total (self-report)			
	Unstandardized coefficient		Standardized coefficient	*p*	Unstandardized coefficient		Standardized coefficient	*p*
	*B*	Standard error			*B*	Standard error		
1. Univariate predictors entered in regression								
VR PTSD (alone)	0.58	0.09	.58	<.001	0.64	0.12	.49	<.001
Initial score for outcome measure (alone)	0.65	0.07	.69	<.001	0.78	0.08	.70	<.001
2. Predictors entered simultaneously								
VR PTSD (allowing for initial outcome measure score)	0.24	0.10	.24	.016	0.13	0.12	.10	.276
Initial score for outcome measure (allowing for VR PTSD symptoms)	0.51	0.09	.53	<.001	0.71	0.11	.64	<.001
*Note.* PTSD = posttraumatic stress disorder; VR = virtual reality; PSSI = Interviewer version of the PTSD Symptom Scale; PDS = Posttraumatic Diagnostic Scale.								
